# In Silico Identification and Molecular Mechanism of Novel Tyrosinase Inhibitory Peptides Derived from Nacre of *Pinctada martensii*

**DOI:** 10.3390/md22080359

**Published:** 2024-08-07

**Authors:** Fei Li, Haisheng Lin, Xiaoming Qin, Jialong Gao, Zhongqin Chen, Wenhong Cao, Huina Zheng, Shaohe Xie

**Affiliations:** 1 College of Food Science and Technology, National Research and Development Branch Center for Shellfish Processing (Zhanjiang), Guangdong Provincial Key Laboratory of Aquatic Products Processing and Safety, Guangdong Provincial Engineering Technology Research Center of Seafood, Guangdong Province Engineering Laboratory for Marine Biological Products, Guangdong Ocean University, Zhanjiang 524088, China; 2112203055@stu.gdou.edu.cn (F.L.); qinxm@gdou.edu.cn (X.Q.); gaojl@gdou.edu.cn (J.G.); chenzhongqin@gdou.edu.cn (Z.C.); cwenhong@gdou.edu.cn (W.C.); zhenghn@gdou.edu.cn (H.Z.); 2 Shenzhen Institute of Guangdong Ocean University, Shenzhen 518108, China; 3 Collaborative Innovation Center of Seafood Deep Processing, Dalian Polytechnic University, Dalian 116034, China; 4 Guangdong Shaohe Pearl Co., Ltd., Shantou 515041, China; xsh5760288@126.com

**Keywords:** *Pinctada martensii*, molecular docking, B16F10 cells, melanogenesis, antioxidant

## Abstract

Pearl and nacre powders have been valuable traditional Chinese medicines with whitening properties for thousands of years. We utilized a high-temperature and high-pressure method along with compound enzyme digestion to prepare the enzymatic hydrolysates of nacre powder of *Pinctada martensii* (NP-PMH). The peptides were identified using LC–MS/MS and screened through molecular docking and molecular dynamics simulations. The interactions between peptides and tyrosinase were elucidated through enzyme kinetics, circular dichroism spectropolarimetry, and isothermal titration calorimetry. Additionally, their inhibitory effects on B16F10 cells were explored. The results showed that a tyrosinase-inhibitory peptide (Ala-His-Tyr-Tyr-Asp, AHYYD) was identified, which inhibited tyrosinase with an IC_50_ value of 2.012 ± 0.088 mM. The results of the in vitro interactions showed that AHYYD exhibited a mixed-type inhibition of tyrosinase and also led to a more compact enzyme structure. The binding reactions of AHYYD with tyrosinase were spontaneous, leading to the formation of a new set of binding sites on the tyrosinase. The B16F10 cell-whitening assay revealed that AHYYD could reduce the melanin content of the cells by directly inhibiting the activity of intracellular tyrosinase. Additionally, it indirectly affects melanin production by acting as an antioxidant. These results suggest that AHYYD could be widely used as a tyrosinase inhibitor in whitening foods and pharmaceuticals.

## 1. Introduction

*Pinctada martensii* is found in mainland China and the South China Sea. Its primary use is in pearl production, making it a significant marine cultural shellfish resource in China [1]. Shells and pearls are similar in structure and material composition. Both are biominerals formed under the regulation of an organic matrix secreted by the outer coat membrane tissue [2]. The nacre is secreted by the outer mantle tissue and consists of a lustrous material that gradually envelops the foreign matter to form a pearl over time [3]. The Chinese Pharmacopoeia 2020 stipulates that the source of medicinal pearls is from the pearl shellfish family, which includes pearl shellfish and other bivalves stimulated by the formation of pearls, and it has been recorded to have the effect of calming the mind and nerves, detoxifying the muscles, moisturizing the skin, and removing blemishes [4]. Pearls and nacre are rich sources of calcium, as well as shell proteins, small amounts of trace metals, hydrophobic amino acids, and aromatic amino acids. Calcium constitutes more than 90% of the weight of the pearl and nacre [5]. Studies have indicated that peptides inhibiting tyrosinase typically consist of these amino acids [6]. Therefore, nacre peptides are considered to have the potential of being tyrosinase-inhibiting peptides. Studies have shown that the main components of nacre powder are extremely similar to those of pearl powder and exhibit a variety of biological activities. Sedative-hypnotic [5], anti-photoaging [6], antioxidant [7], trauma repair [8], are some of the functional activities of pearl powder as supported by modern medicine. However, the specific whitening ingredients and mechanisms remain unclear.

Excessive melanin deposition can lead to a range of skin problems. Melanin is a phenolic polymer widely distributed in plants and animals [9]. It is the main factor in determining the color of the skin and hair and can inhibit ultraviolet rays and free radicals that stimulate damage to the skin cells [10]. However, when melanin is over-synthesized, excessive deposition of melanin in the surface layer of the skin may result in a series of skin diseases such as dark spots, brown spots, and further promote skin aging; ultimately, this excessive melanin production could lead to melanoma and other skin cancers. Tyrosinase is a copper-containing metal oxidase and is the key enzyme that regulates melanin production. Tyrosinase oxidizes tyrosine to produce dopamine quinone, which reacts with amino acids or proteins, ultimately resulting in melanin production [11]. Therefore, tyrosinase inhibition and antioxidants are particularly crucial. There are two main ways to inhibit tyrosinase: one involves direct inhibition of tyrosinase, while the other involves indirect regulation of the antioxidant system to inhibit tyrosinase production [12,13]. Currently proven effective whitening ingredients, such as Kojic acid and its derivatives, Resveratrol and Arbutin, have been found to have a stronger inhibitory effect on tyrosinase [14,15,16]. However, they are also prone to side effects such as skin redness, itching, ulcers, and even toxicity to the body [17,18]. The current whitening products available on the market, while commonly used, still carry a higher risk of causing allergic reactions or other adverse effects on the user’s skin [19,20]. Therefore, safe and stable whitening preparations are receiving increasing attention. A large number of by-products, such as shells, are produced during the pearl harvest from the *Pinctada martensii* [21]. The main ways of utilization include decoration, building paint, feed, and other purposes. Shell meat, as well as other tissues, has been the main focus of existing studies, while the active peptides in the inner nacre of the shell have been less studied. Previous studies have shown that peptides extracted from the shell flesh tissues of pearl oysters and their related tissues have demonstrated a variety of biological activities, such as antioxidant [22], anti-photoaging [23], and blood pressure-lowering effects [24]. Zhou et al. simulated gastrointestinal hydrolysis to prepare peptides from the meat of *Pinctada martensii* and verified the antioxidant activity of the peptides using HepG_2_ cells [22]. A study successfully predicted potential tyrosinase-inhibitory peptides from abalone proteins using an anti-tyrosinase prediction tool [25]. However, there has been limited research on potential whitening peptides within the shell of pearl oysters.

In the present study, peptides derived from the nacre powder of *Pinctada martensii* after undergoing high temperature and pressure treatment. Neutral proteases have been used in the enzymatic digestion of shellfish. To break down the insoluble proteins in the pearl layer, we conducted a combination of neutral proteases and pineapple protease for enzymatic digestion, and then characterized them using LC–MS/MS. The potential tyrosinase-inhibitory peptides were then screened through molecular docking. Subsequently, they were synthesized in solid phase and examined for their tyrosinase-inhibitory mechanism and antioxidant capacity at both in vitro and cellular levels. These findings could contribute to the development of new cosmetic ingredients and natural food-based functional foods.

## 2. Results and Discussion

### 2.1. Inhibition of Tyrosinase and Antioxidant Activity of the Enzymatic Hydrolysis Product

A preliminary investigation of tyrosinase activity and antioxidant activity was conducted on the enzymatic hydrolysis product obtained from nacre powder after high-temperature and high-pressure treatment and complex enzymatic digestion. The results, as presented in Table 1, indicate that the nacre peptide digest exhibits a certain inhibitory effect on tyrosinase and possesses antioxidant properties. These findings suggest that it holds promise for the development of tyrosinase-inhibiting peptides.

### 2.2. Identification and Molecular Docking

The mass spectrometry data were analyzed using the software PEAKS Studio 8.5. The identification results were compared using the UniProt database. For efficient screening, peptides with amino acid residues < 10 and score > 20 were selected to docking with tyrosinase (2Y9X) [26].

Molecular docking is a commonly used method to simulate the way molecules interact with each other and predict their binding modes and affinities using a computerized platform [27]. The method is widely used to probe the binding ability of small molecules interacting with large molecules [28]. A lower intermolecular binding energy indicates better peptide–protein affinity and theoretically more tyrosinase-inhibitory activity of the peptides.

**Figure 1:**
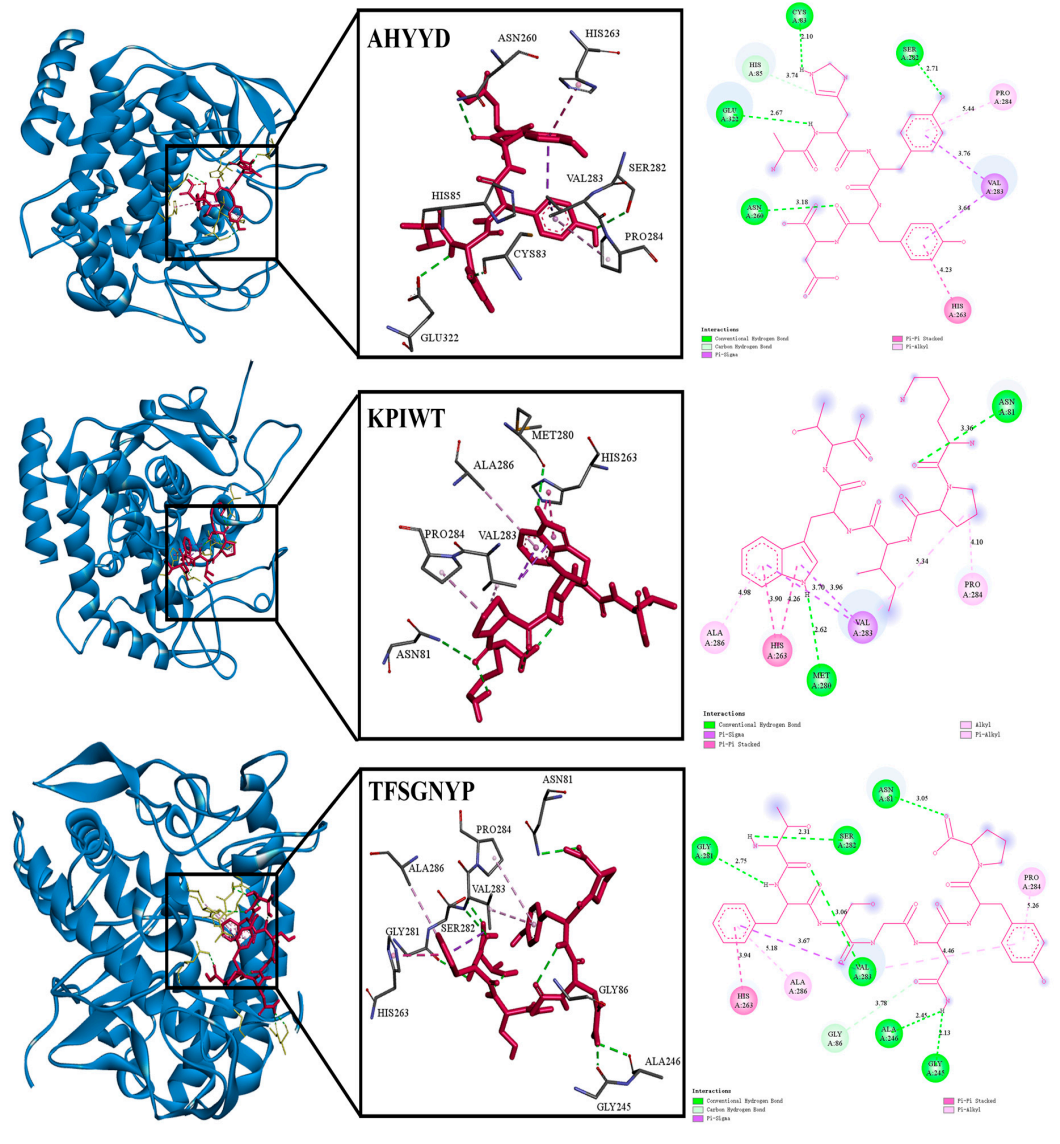
The 3D and 2D visualizations of molecular docking of AHYYD, KPIWT, and TFSGNYP with tyrosinase (2Y9X).

As shown in Table 2, molecular docking of 32 peptides was performed using Autodock vina, and the receptor was tyrosinase [29]. In terms of amino acid composition, hydrophobic amino acids, aromatic ring-containing amino acids, and polar amino acids play a crucial role in protein–ligand interactions [30]. Additionally, the amino acid composition also influences the water solubility of peptides. Taking into account the peptide’s GRAVY score < −0.5, amino acid composition, and binding energy, three peptides—AHYYD, KPIWT, and TFSGNYP—were finally selected for the subsequent study. The predicted binding energies of AHYYD, KPIWT, and TFSGNYP were −8.0 kcal/mol, −7.4 kcal/mol, and −7.3 kcal/mol, respectively. Docking binding was slightly lower than the results for peptides extracted from *Atrina pectinata* Mantle, where a lower binding energy indicates a more favorable binding effect [31]. To visually demonstrate how the peptides bind to tyrosinase, a visual analysis was conducted using the Discovery Studio 2019 client tool. As shown in Table 3 and Figure 1, Docking results showed that the peptide formed interactions with residues such as HIS263, ASN260, and VAL283 near the active site pocket of tyrosinase [32]. AHYYD formed four hydrogen bonds with SER282, CYS83, GLU322, and ASN260 of tyrosinase. The bond lengths ranged from 2.10 Å to 3.18 Å. KPIWT formed two hydrogen bonds with MET280 and ASN81 of tyrosinase, with bond lengths of 2.62 Å and 3.36 Å. TFSGNYP formed six hydrogen bonds with ASN81, SER282, GLY281, VAL283, ALA246, and GLY245 of tyrosinase, and the bond lengths ranged from 2.13 to 5.26 Å. The shorter the hydrogen bond, the greater the bond energy, indicating that the ligand and receptor form a more stable bond [26]. In the hydrophobic interactions with tyrosinase, all three peptides form hydrophobic interactions with PRO284 and HIS263. KPIWT and AHYYD form hydrophobic interactions with VAL283. KPIWT and TFSGNYP form hydrophobic interactions with ALA286. On the basis of the amino acid residues where the three peptides form hydrophobic interactions, most of the amino acids they interact with are similar to each other. Due to the similarity of the hydrophobic interactions, we infer that the difference in binding energies is primarily due to variations in hydrogen bonding. Hydrogen bonding is identified as the main driving force.

### 2.3. Molecular Dynamics Simulation

**Figure 2:**
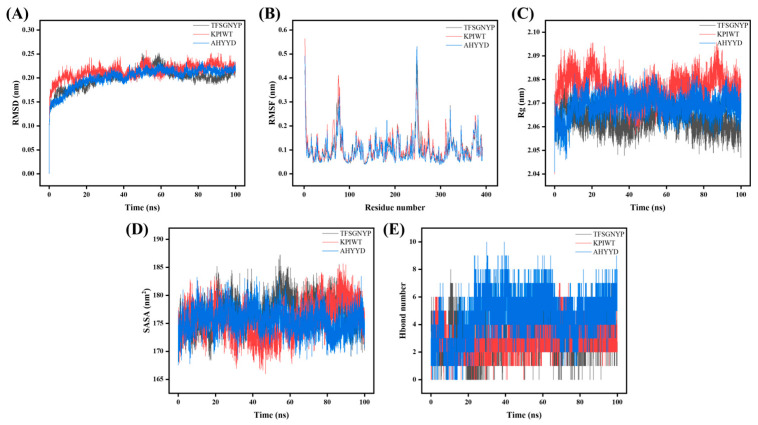
Molecular dynamics results of tyrosinase with AHYYD, KPIWT, and TFSGNYP. (**A**) RMSD; (**B**) RMSF; (**C**) Rg; (**D**) SASA; (**E**) Hbond number.

The stability and binding affinity between tyrosinase and the three small molecules were further investigated using the GROMACS program, with the three complex docked systems as the initial structure. The 100 ns molecular dynamics (MD) simulation method was used to obtain stable complex systems for further comparison of the three small molecules at the molecular level. The trajectory data obtained from the 100 ns were analyzed to derive the kinetic properties of the three complexes. Root mean square deviation (RMSD), radius of gyration (Rg), solvent accessible surface area (SASA), root mean square fluctuation (RMSF), and Hbond number were utilized to assess each system in the MD study, and the results are presented. As shown in Figure 2, a graph of each parameter of the dynamics is plotted.

Root mean square deviation (RMSD) is a metric used to assess structural changes in proteins [33]. The results show that the RMSD between proteins and small molecules is large in the first 30 ns of the simulation. Tyrosinase with AHYYD stabilizes the fastest, the RMSD value remains within a small range, indicating that the binding of proteins to small molecules is relatively stable. Root mean square fluctuation (RMSF) is a metric used to assess the dynamics of proteins [34]. The results show that the residues in the key regions increase in flexibility, and the RMSF values are larger in the binding portion in the 300–400 region. Tyrosinase with AHYYD and tyrosinase with TFSGNYP were more variable. The RMSF of tyrosinase with AHYYD reaches a maximum value of 0.5 nm, while it is smaller in the unbound portion. The RMSF values are all below 1 Å. The minimal fluctuation indicates that these atoms create stable complexes with tyrosinase because of robust intermolecular interactions, restricting their movement in molecular dynamics simulations [35]. Gyrate is an indicator of the overall compactness of a protein and characterizes the distribution of system atoms along a specific axis [28]. The results indicate that after the binding of the protein with the three small molecules, the gyrate value of tyrosinase with AHYYD tends to stabilize after 20 ns, while the gyrate value of tyrosinase with TFSGNYP tends to decrease after 60 ns. This suggests that the binding of small molecules leads to a more compact protein structure. KPIWT, on the other hand, kept fluctuating within a small range repeatedly. SASA is a metric used to assess the surface area of proteins. The results indicate that the amplitude of the tyrosinase with AHYYD curve fluctuates slightly between 170 and 180 and then decreases slightly. This suggests that protein–protein interactions have little effect on the stability of protein molecules. The amplitudes of the tyrosinase with TFSGNYP and tyrosinase with KPIWT curves are similar, but they exhibit opposite trends. The SASA value of tyrosinase with TFSGNYP shows a decreasing trend after 60 ns, indicating that the binding of small molecules causes the protein molecule to become more compact, aligning with the results of gyrate analysis. A hydrogen bond is a metric used to assess hydrogen bonding between proteins and small molecules. The results show that many hydrogen bonds are formed between proteins and small molecules during the simulation. These bonds are dominated by interactions between key residues in tyrosinase and peptides in the molecular docking results.

Analyzing the RMSD, RMSF, Gyrate, SASA, and Hbond number results of the three peptide–enzyme complexes mentioned above, it can be concluded that AHYYD and TFSGNYP interact with tyrosinase more effectively than KPIWT, protein–small molecule interaction appears stable, and the binding of small molecules leads to a more compact protein structure.

### 2.4. Tyrosinase-Inhibitory Activity and Antioxidant Capacity of Synthetic Peptides

In terms of amino acid composition, AHYYD contains two repetitive benzene-ring-containing amino acid residues, Tyr, and a hydrophobic amino acid, Ala. It has been shown that the repetitive amino acid sequence enhances tyrosinase inhibition [36]. TFSGNYP contains the polar amino acid Ser, a benzene-ring-containing amino acid residue, Phe, and a hydrophobic amino acid, Pro. KPIWT has the benzene-ring-containing amino acid residue Trp and the hydrophobic amino acid Pro. It has been reported that hydrophobic amino acids, benzene-ring-containing amino acid residues, and polar amino acids contribute to tyrosinase inhibition. As shown in Table 4, the synthetic peptides AHYYD, KPIWT, and TFSGNYP derived from *Pinctada martensii* exhibited varying degrees of inhibitory activity against tyrosinase, with Kojic acid serving as a positive control. From the molecular docking visualization results, it can be seen that although the hydrophobic interactions between the three peptides and the enzyme are relatively similar, their distinct hydrogen-bonding interactions may affect the inhibition of tyrosinase activity in vitro. Among these, AHYYD demonstrated a significant inhibitory effect, its IC_50_ value is 2.012 ± 0.088 mM, consistent with the findings of molecular docking screening; this result is similar to the peptides activity identified in the mantle of *Atrina pectinata* [31].

**Figure 3:**
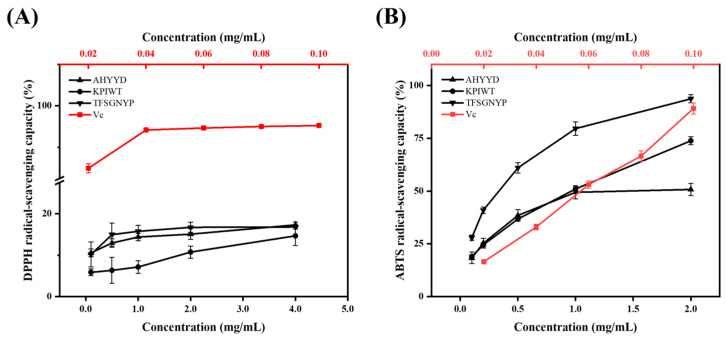
Scavenging capacity of peptides for DPPH free radicals (**A**); Scavenging capacity of peptides for ABTS free radicals (**B**).

As depicted in Figure 3, there was no significant difference in DPPH clearance between AHYYD and TFSGNYP at any of the five concentrations (*p* > 0.05). However, KPIWT showed a significant difference from both AHYYD and TFSGNYP at concentrations of 0.1–2 mg/mL; AHYYD and TFSGNYP exhibited a stronger DPPH clearance effect than KPIWT (*p* < 0.05) at the corresponding concentrations. There was no significant difference in the clearance of DPPH by the three peptides at a concentration of 4 mg/mL. The IC_50_ values for ABTS clearance by AHYYD, KPIWT, and TFSGNYP were 1.416 mg/mL, 0.7955 mg/mL, and 0.2785 mg/mL, respectively, with a significant difference between the three peptides (*p* < 0.05). A smaller IC_50_ value indicates a better scavenging effect. It can be observed that TFSGNYP exhibits the strongest scavenging ability for ABTS radicals, followed by KPIWT and AHYYD. Vitamin C (Vc) was utilized as a positive control.

### 2.5. Inhibition Dynamics Analysis

**Figure 4:**
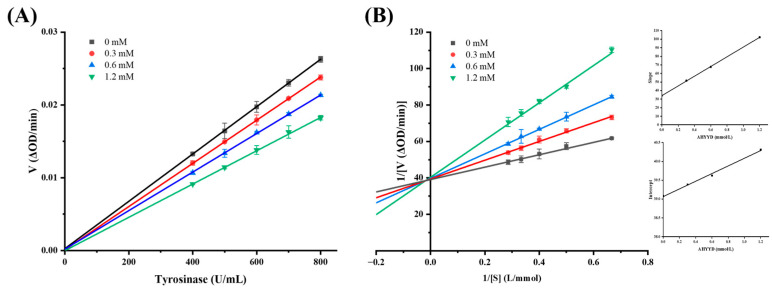
(**A**) Plots of enzymatic reaction rate versus tyrosinase concentration. (**B**) Plots of Lineweaver–Burk; the secondary plots of slope and Y-intercept versus concentration of AHYYD are shown in the inset.

Based on the observation that the inhibition of tyrosinase by the synthetic peptide AHYYD was reversible, the type of inhibition was further elucidated by constructing a Lineweaver–Burk double inverse plot of the enzymatic reaction rate against the concentration of the substrate (L-tyrosine). In this plot, the intercept with the Y-axis represents 1/Vmax, while the intercept with the X-axis represents −1/Km. As depicted in Figure 4, when the concentration of tyrosinase, the concentration of synthetic peptide (0, 0.3, 0.6, 1.2 mM, respectively), and the concentration of substrate L-tyrosine remained constant, the reciprocal of the enzymatic reaction rate exhibited a linear correlation with the reciprocal of the substrate concentration. The slope of the line increased with the rise in synthetic peptide concentration, indicating a mixed type of inhibition [37]. By using the quadratic plotting method, the slopes of the four straight lines in the Lineweaver–Burk double inverse plot were plotted against the synthetic peptide concentration [38]. The inhibition constants of AHYYD on the free enzyme were calculated as K_i_ = 0.601 mmol/L, and as K_is_ = 38.375 mmol/L on the enzyme–substrate complex. The smaller the inhibition constants, the higher the binding affinity of the inhibitor to the enzyme, K_i_ << K_is_ indicates that AHYYD clearly favors the binding site with the free enzyme.

### 2.6. CD Spectra Analysis

**Figure 5:**
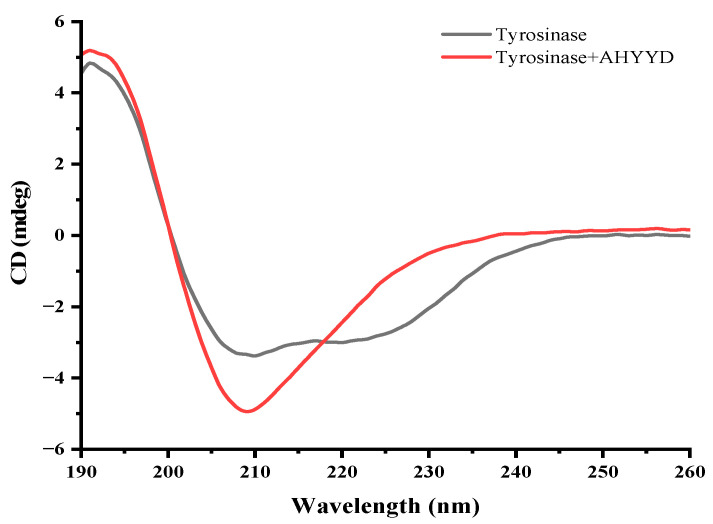
The CD spectra of tyrosinase in the absence and presence of AHYYD. c (tyrosinase) = 0.4 mg/mL, and c (AHYYD) = 0.6 mM.

CD spectroscopy is a precise method used to study the secondary structure of biomolecules, including proteins. It allows for the rapid analysis of protein conformational changes caused by the addition of ligands [39]. The addition of AHYYD caused changes in the secondary structure of tyrosinase (Figure 5). In the spectrum of free tyrosinase, there are two negative bands near 208 nm and 222 nm, which are characteristic peaks of the α-helical structure in the secondary structure [40]. The content of α-helix, β-folding, and β-turning increased from 32.53%, 16.27%, and 18.20% to 36.8%, 23.77%, and 21.93% and the random coil content decreased from 30.83% to 18.33%, respectively. The rise in the α-helix content suggests that the interaction between the peptide and tyrosinase caused the tyrosinase structure to become more compact. This finding contrasts with the interaction between tretinoin and tyrosinase, suggesting that it leads to a change in the tyrosinase conformation to some extent [37].

### 2.7. Isothermal Titration Calorimetry Analysis

**Figure 6:**
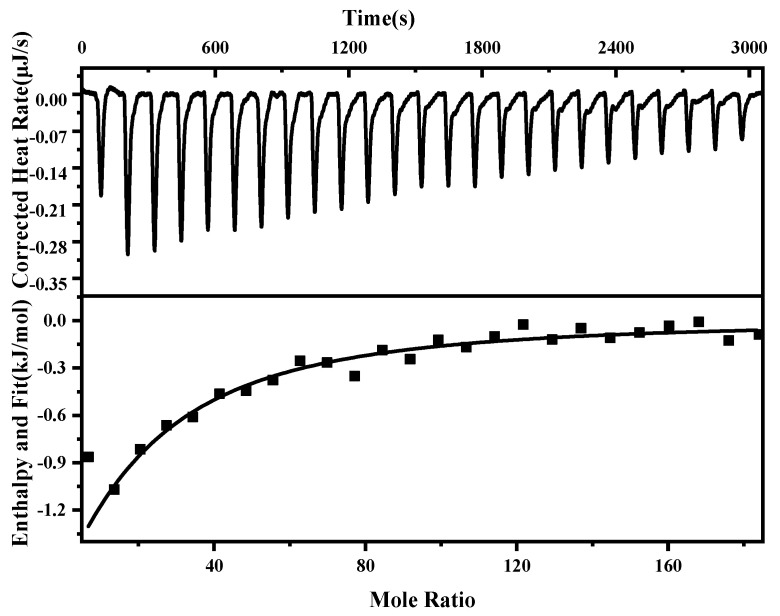
Interaction of AHYYD with tyrosinase studied by ITC at 37 °C showed the thermogram and binding isotherm (tyrosinase) = 0.001 mM, and c (AHYYD) = 1 mM.

ITC can measure the binding strength of protein–protein interactions by quantifying the thermodynamic changes in complex reactions [41]. The titration of tyrosinase by AHYYD exhibited significant heat changes, indicating binding (Figure 6). The data were analyzed using the independent model, and the thermodynamic binding parameters were calculated. The results showed that the enthalpy (∆H) was −7.282 kJ/mol, the binding constant (Ka) was 2.495 × 10^4^ M^−1^, the entropy change (∆S) was 60.7 J/mol·K, and the standard free energy change (∆G) was −1.883 × 10^4^ kJ/mol. Negative values of ∆G indicate that the bimolecular reaction is spontaneous. The change in enthalpy indicates that the process is essentially exothermic, which is consistent with the findings of Yu et al [34]. The titration curves are more consistent with the “one set of binding sites” model [42]. The mechanism of inhibition categorizes AHYYD as competitive inhibition, indicating that AHYYD binds to the active site of tyrosinase. This finding further confirms the proposed mechanism of inhibition based on inhibition kinetics.

### 2.8. Study on the Whitening Effect of Peptides Based on B16F10 Cells

#### 2.8.1. Cell Viability

**Figure 7:**
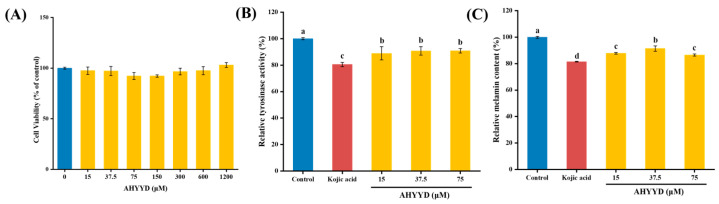
(**A**) Effects of AHYYD on cell viability. (**B**) Effects of AHYYD and Kojic acid on tyrosinase activity in B16F10 cells. (**C**) Effects of AHYYD and Kojic acid on melanin production in B16F10 cells. c (Kojic acid) = 350 μM. Different letters indicate that there are significant differences in data (*p* < 0.05).

As depicted in Figure 7A, there was no significant difference in the viability of B16F10 cells within the concentration range of 15–1200 μM concentration of the synthetic peptide AHYYD compared to the control group (*p* > 0.05). This suggests that the synthetic peptide AHYYD did not exhibit any cytotoxic effects. Consequently, the concentrations of 15, 37.5, and 75 μM were chosen for the subsequent experiments.

#### 2.8.2. Effect of AHYYD on the Tyrosinase Activity and Melanin Content of B16F10 Cells

As shown in Figure 7B,C, compared with the control group, AHYYD significantly inhibited the tyrosinase activity of B16F10 cells in the concentration range of 15–75 μM (*p* < 0.05). Additionally, the intracellular melanin content was significantly reduced by AHYYD in the concentration range of 15–75 μM (*p* < 0.05). The trend in tyrosinase activity and melanin content mirrored that of the peptides extracted from pearl shell meat [26]. At the maximum treatment concentration, the intracellular tyrosinase activity and melanin content were reduced by 9.08% and 13.48%, respectively. Kojic acid caused a reduction of 19.51% in tyrosinase activity and 18.54% in melanin content.

#### 2.8.3. Effect of AHYYD on the Antioxidant Enzyme Activity and ROS Content of B16F10 Cells

**Figure 8:**
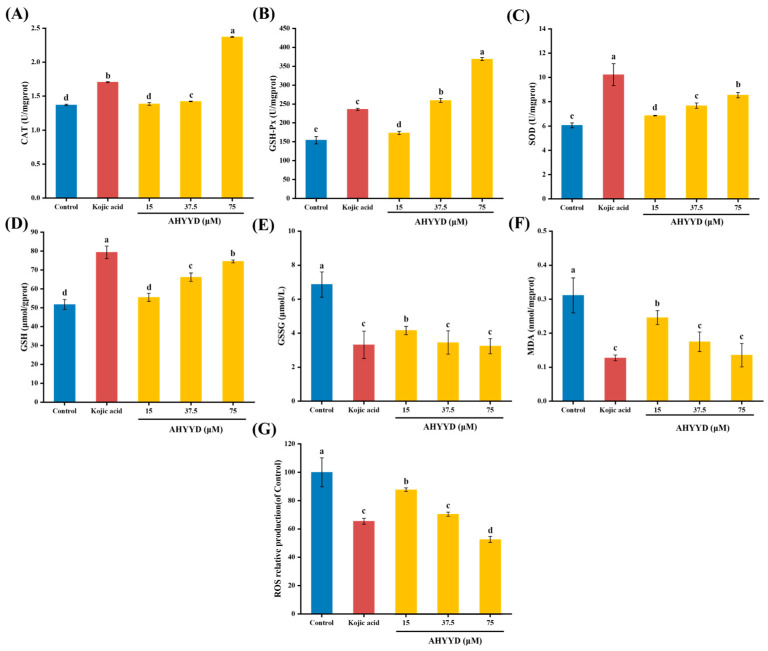
Effect of AHYYD on the intracellular antioxidant capacity in B16F10 cells; (**A**) CAT; (**B**) GSH-Px; (**C**) SOD; (**D**) GSH; (**E**) GSSG; (**F**) MDA; (**G**) ROS. c (Kojic acid) = 350 μM. Different letters indicate that there are significant differences in data (*p* < 0.05).

SOD, CAT, and GSH-Px are antioxidant enzymes that collaborate to regulate antioxidant processes and safeguard cells from oxidative stress damage [43]. As can be seen from Figure 8, the activities of GSH-Px and SOD enzymes exhibited a concentration-dependent relationship with increasing concentrations of AHYYD. The activities of CAT, GSH-Px, and SOD increased by 73.05%, 139.08%, and 41.25%, respectively, under the maximum treatment concentration, and by 24.55%, 52.85%, and 68.92%, respectively. At the maximum treatment concentration, the CAT and GSH-Px activities were superior (*p* < 0.05) compared to Kojic acid. However, both of them were still far away from Kojic acid in terms of SOD activity (*p* < 0.05). Therefore, AHYYD exhibited a protective effect on the antioxidant system by enhancing the activity of antioxidant enzymes in B16F10 cells, thereby preventing melanin production. Reactive oxygen species (ROS) serve as a crucial indicator of antioxidant connectivity in the organism. As shown in Figure 8G, at a concentration of 75 μM, the intracellular ROS levels were reduced to 52.61% and 65.44% of the untreated group after AHYYD and tretinoin treatment, respectively.

GSH, GSSG, and MDA are crucial metabolic regulators in the cell [44]. GSH helps maintain normal immune system function and has antioxidant effects. It exists in two forms: reduced glutathione (GSH) and oxidized glutathione (GSSG). MDA content is a significant parameter that reflects the body’s antioxidant potential, indicating the rate and intensity of lipid peroxidation and indirectly reflecting the extent of tissue peroxidation damage. Under the maximum treatment concentration, the content of GSH increased by 44.07%, while the content of GSSG and MDA decreased by 52.86% and 56.56%, respectively. These changes were not significantly different from those observed in the positive control group treated with Kojic acid (*p* > 0.05). This suggests that AHYYD demonstrates a superior ability in regulating GSH, GSSG, and MDA levels. Along with the antioxidant enzymes mentioned above, it effectively protects B16F10 cells from oxidative damage.

## 3. Materials and Methods

### 3.1. Materials

The nacre powder of the *Pinctada martensii* was purchased from Guangdong Shaohe Pearl Co., Ltd. (Shantou, China). The tyrosinase (from Mushroom, 500 U/mg) and PBS buffer were purchased from Shanghai Yuanye Biotechnology Co., Ltd. (Shanghai, China). The L-tyrosine was obtained from Shanghai Macklin Biochemical Technology Co., Ltd. (Shanghai, China). The fetal bovine serum (FBS) was purchased from Serana Europe GmbH (Dorfstrasse 17A, Pessin, Brandenburg, Germany), and the penicillin–streptomycin mixture was purchased from Beijing Soleberg Biotechnology Co., Ltd. (Beijing, China). The DMEM/F−12 medium and trypsin-EDTA digest were purchased from Thermo Fisher Scientific (Shanghai, China).

### 3.2. Preparation of Enzymatic Digests of Nacre Peptides

A total of 50 g of nacre powder from *Pinctada martensii* was dissolved in 250 mL (*v:w* = 5:1) of water and heated at high temperature (121 °C) and pressure for 20 min. Then, 0.1250 g of pineapple protease (0.25% by mass of nacre powder) and 0.2500 g of neutral protease (0.5% by mass of nacre powder) were added to the pre-treated nacre powder solution, and the enzyme digestion was conducted at a temperature of 50 °C for 4 h. Finally, the mixture was heated at 100 °C for 20 min. The supernatant was centrifuged at 8000 rpm for 20 min at 4 °C to obtain the pearl peptide solution after vacuum concentration.

### 3.3. Tyrosinase-Inhibitory Activity and Antioxidant Activity Assay of Enzyme Digests

We followed the method of Yu [37], with a slight modification. L-tyrosine solution (0.5 mg/mL), sample solution, and PBS buffer were sequentially added into the 96-well plate, thoroughly mixed, and then incubated at 37 °C for 10 min, then 20 μL of tyrosinase solution (500 U/mL) was added into each well, followed by immediate placement into the enzyme marker after the reaction at 37 °C for (10 min ± 5 s). The results were analyzed at 475 nm. DPPH and ABTS free-radical-scavenging capacity assays were conducted using kits from Grace Biotechnology Co., Ltd. (Jiangsu, China).

(1) $$Tyrosinase\;inhibition\;rate\%=\left(1-\frac{A_{4}-A_{3}}{A_{2}-A_{1}}\right)\times 100\%$$

where A_4_ is the absorption value of the reaction well, A_3_ is the absorption value of the Sample Background well, A_2_ is the absorption value of the Solvent reaction well, and A_1_ is the absorption value of the Solvent base well.

### 3.4. Protein Sequence Identification

The amino acid sequence was identified by using the Bio-Tech Pack Technology Co. (Beijing, China). The nacre peptides were reduced and alkylated separately as samples, an Easy-nLC 1200 system coupled with a Q Exactive™ Hybrid Quadrupole-Orbitrap™ Mass Spectrometer (Thermo Fisher Scientific, Waltham, MA, USA) with an ESI nanospray source. Then, they were analyzed by liquid chromatography–mass spectrometry (LC–MS/MS) to generate a raw file of the mass spectrometry results. The total ion chromatograms were obtained using Xcalibur 4.0 software. The mass spectrometry data were analyzed using the software PEAKS Studio 8.5, and the identification results were compared with the UniProt database.

### 3.5. Molecular Docking Studies

Studying the peptides and tyrosinase using molecular docking methods, we referred to the method of Wang [31]. The ligands and proteins required for molecular docking were prepared using AutoDock Vina 1.1.2 software. Tyrosinase (PDB: 2Y9X) was utilized as the receptor, and a peptide was employed as the ligand. The peptides were designed using ChemDraw and energy-minimized using Chem3D. The crystal structure of the target protein was obtained from the PDB database (https://www.rcsb.org/structure/2Y9X, accessed on 25 November 2023). Autodock Vina 1.1.2 software was used to perform molecular docking with default parameters to calculate the binding energy of the peptide sequence to the enzyme. The binding-free energy (kcal/mol) of the target structure represents the binding ability of the two molecules, and the lower the binding-free energy, the more stable the binding between the ligand and the receptor. Pymol (https://pymol.org/2/, accessed on 3 December 2023) was used for visual analysis, while Discovery Studio 2019 was utilized for further exploration. Discovery Studio 2019 (https://www.3ds.com/products/biovia/discovery-studio/visualization, accessed on 3 December 2023) was used for visual analysis of the 2D images. The tyrosinase docking centers were X = −10.09, Y = −28.03, and Z = −43.14.

### 3.6. Molecular Dynamics Simulations

GROMACS 2022.3 software was used for the molecular dynamics simulation [45,46]. For small molecule preprocessing, AmberTools22 was employed to apply the GAFF force field to small molecules, while Gaussian 16W was used for hydrogenating small molecules and calculating the RESP potential. Potential data will be added to the topology file of molecular dynamics system. Following the completion of the simulation, the software’s built-in tool was utilized to analyze the trajectory. The root mean square variance (RMSD), root mean square fluctuation (RMSF), solvent accessible surface area (SASA), radius of gyration (Rg), and hydrogen bond were calculated for each amino acid trajectory.

### 3.7. Peptides Synthesis

The peptide was obtained through solid-phase synthesis and synthesized by Aminolink Biotechnology Co., Ltd. (Shanghai, China). Three peptides with purity above 98% (*w*/*w*) were detected by HPLC: Ala-His-Tyr-Tyr-Asp (AHYYD), Thr-Phe-Ser-Gly-Asn-Tyr-Pro (TFSGNYP), and Lys-Pro-Ile-Trp-Thr (KPIWT). Mobile phase A, 0.1% trifluoroacetic in 100% water; mobile phase B, 0.1% trifluoroacetic in 100% acetonitrile; flow rate, 1 mL/min; column, Inertsil ODS-SP (4.6 × 250 mm × 5 µm), with a detection wavelength of 220 nm. Eventually, the purified peptides were identified by ESI–MS spectroscopy.

### 3.8. Determination of Inhibition Type

Keeping the L-tyrosine concentration constant at 0.5 mg/mL, the concentrations of the synthetic peptide and tyrosinase were varied. The change in the OD_475_ absorbance value was continuously monitored at 37 °C after mixing. The enzyme concentration is plotted on the horizontal axis, while the rate of absorbance change is plotted on the vertical axis. Under the conditions of varying the peptide concentration and substrate concentration while keeping the tyrosinase solution constant, the reciprocal of the substrate concentration was plotted on the horizontal axis, and the reciprocal of the rate of absorbance change was plotted on the vertical ax.

### 3.9. CD Measurements

We used circular dichroism to study peptide–enzyme interactions, referring to the method of Yu [47]. The concentration of AHYYD was 0.6 mM, and the concentration of tyrosinase was 0.4 mg/mL. The reaction system comprised 100 μL of tyrosinase solution mixed with 20 μL of AHYYD solution, which reacted for over 30 min at 37 °C. All data were collected three times. The changes in the secondary structure were analyzed using CDNN 2.1 software.

### 3.10. Isothermal Titration Calorimetry Analysis

Peptide (1 mM) was used to titrate tyrosinase (0.001 mM) to study thermodynamic changes. The initial volume of the peptide solution for titration was 50 μL (inhaled into the syringe); the initial volume of the tyrosinase solution for titration was 300 μL (added to the cuvette). The reaction conditions included adding 25 drops of 2 μL each with a 120 s interval between drops at 37 °C and a stirring speed of 350 r/min; the experimental group involved titrating the peptide with tyrosinase, while the control group involved titrating the peptide with a solvent buffer. We calculated the binding constant (Ka), enthalpy change (∆H), and entropy change (∆S) during the Gibbs free energy (∆G) changes [39].

(2) ∆G=∆H−T·∆S

### 3.11. Cytotoxicity of AHYYD to B16F10 Cells

The B16F10 cells were obtained from FengHui Biotechnology Co., Ltd. (Hunan, China). B16F10 cell cultures from generations 10–25 were grown in medium (DMEM supplemented with 10% fetal bovine serum, 1% antibiotic–antifungal solution) in a 5% CO_2_ incubator at 37 °C.

Cell viability was assessed using the Cell Counting Kit-8 (CCK-8) assay. During the logarithmic growth phase, 100 µL of cell suspension was dispensed into 96-well plates, with 3000 cells per well, and the supernatant was removed after incubation at 37 °C for 24 h. Wells containing 100 µL of medium served as blank controls. Determination of viability after 48 h of sample treatment.

### 3.12. Effect of AHYYD on Melanin and Tyrosinase Synthesis in B16F10 Melanoma Cells

The cells in the logarithmic growth phase were seeded at a density of 2 × 10^5^ cells per well in 6-well plates. After 24 h of culture, the supernatant was removed. The control group, positive control group for Kojic acid (350 μM) [48], and peptide-treated groups (15, 37.5, 75 μM) were established. After 48 h of incubation, the cells were lysed, and the cell lysates were centrifuged at 12,000 rpm for 10 min. The reaction mixture consisted of 50 µL of cell lysate supernatant, 50 µL of 1 mg/mL L-DOPA, and 50 µL of PBS buffer. The mixture was incubated at 37 °C for 1 h. The dopamine formation level was then measured spectrophotometrically at 475 nm. Tyrosinase activity was expressed as a percentage of the blank control [49].

The cell culture stage for melanin content determination was as follows: the cells were washed twice with PBS buffer after 48 h of peptide treatment. Trypsin digestion was performed, and the cells were collected. The lower precipitate was removed by centrifugation for 5 min and the precipitate was lysed by dissolving it in 1 mol/L NaOH containing 10% DMSO for 1 h at 80 °C. Subsequently, the cell lysate was centrifuged at 12,000 rpm for 10 min. A total of 200 µL of the supernatant was transferred to a 96-well plate, and the absorbance was measured at 405 nm. The melanin content was then expressed as a percentage of the control group [49].

### 3.13. Effect of AHYYD on Antioxidant Enzyme Activity and ROS in B16F10 Cells

The cell culture and treatment methods were the same as those described in Section 3.12. The activities of catalase (CAT), glutathione peroxidase (GSH-Px), and superoxide dismutase (SOD) were determined using the assay kit from Nanjing Jianjian Bioengineering Research Institute (Nanjing, China). The ROS content was determined using the method provided by Biyuntian Bio-Technology Company Limited (Shanghai, China).

### 3.14. Statistical Analysis

All data are expressed as the mean ± standard deviation (SD) of at least three different experiments. Multiple group comparisons were conducted using one-way analysis of variance (ANOVA) and Duncan’s multiple-range test in IBM SPSS Statistics 27. A *p*-value less than 0.05 was considered statistically significant. The molecular docking was performed using AutoDock Vina, and the molecular dynamics simulations were performed by GROMACS 2022.3. The graphical abstracts were created using Figdraw 2.0.

## 4. Conclusions

In the present study, we found that peptides derived from the nacre powder of the *Pinctada martensii* exhibit significant potential for inhibiting tyrosinase. The inhibitory peptide AHYYD was identified from nacre powder peptide by LC–MS/MS and molecular docking screening. It exhibited tyrosinase inhibition with an IC_50_ value of 2.012 ± 0.088 mM. Inhibition kinetics and isothermal titration results showed that AHYYD was a reversible competitive inhibitor. Molecular docking and molecular dynamics simulations indicated that AHYYD had a strong binding affinity, with a binding energy of −8.0 kcal/mol. The effects of AHYYD on tyrosinase activity, melanin production, and antioxidant enzyme activities were investigated in mouse B16F10 melanoma cells. The results showed that AHYYD significantly inhibited intracellular tyrosinase activity and melanin content, and had a positive effect on the intracellular antioxidant enzyme system. It can be concluded that the peptide AHYYD extracted from *Pinctada martensii* has good therapeutic efficacy. This can be further confirmed through animal experiments and utilized for the development of food or cosmetic products. 

## Figures and Tables

**Table 1:** Tyrosinase-inhibitory activity and antioxidant activity of Enzymatic hydrolysis product NP-PMH.

Enzymatic Hydrolysis Product	Tyrosinase Inhibitory Activity (IC_50_, mg/mL)	DPPH Free Radical Scavenging (1 mg/mL, %)	ABTS Free Radical Scavenging (1 mg/mL, %)
NP-PMH	6.743 ± 0.067	1.139 ± 0.232	85.050 ± 2.770

**Table 2:** Potential tyrosinase-inhibitory peptide sequences: screening by mass spectrometry and molecular docking.

NO.	Sequences	Length	Score	Toxicity	Affinity	Gravy
1	AHYYD	5	27.12	Non	−8.0	−1.5
2	GGFGNW	6	30.94	Non	−7.8	−0.47
3	KPIWT	5	20.49	Non	−7.4	−0.52
4	TFSGNYP	7	21.74	Non	−7.3	−0.79
5	ATFDAI	6	27.86	Non	−7.2	1.12
6	NRIPN	5	25.39	Non	−7.2	−1.72
7	KRSLE	5	21.85	Non	−7.0	−1.78
8	HKDGY	5	26.84	Non	−6.9	−2.46
9	ERHLGY	6	24.12	Non	−6.9	−1.52
10	SIIDEVVA	8	23.32	Non	−6.9	1.43
11	KDLFF	5	20.31	Non	−6.9	0.4
12	GHSLTQF	7	35.06	Non	−6.8	−0.29
13	FGSLSF	6	24.8	Non	−6.8	1.23
14	GGSFSVR	7	26	Non	−6.8	0.01
15	TNNFT	5	20.4	Non	−6.7	−1.12
16	LPEEV	5	23	Non	−6.7	−0.12
17	SASTTLEE	8	22.84	Non	−6.6	−0.55
18	VTANPANT	8	21	Non	−6.6	−0.28
19	HSSAHS	6	20.36	Non	−6.5	−1.17
20	TNTSNP	6	23.65	Non	−6.5	−1.8
21	SDLGGI	6	21.62	Non	−6.5	0.53
22	MVSLEG	6	20.69	Non	−6.5	0.87
23	LKGHEDL	7	20.01	Non	−6.4	−0.99
24	SIDLYK	6	20.7	Non	−6.4	−0.2
25	MDLSHA	6	21.73	Non	−6.3	0
26	DYQLP	5	21.75	Non	−6.2	−1.22
27	KEMQGG	6	20.62	Non	−6.2	−1.63
28	HTLESKPNPD	10	22.99	Non	−5.9	−1.85
29	KEPNK	5	20.77	Non	−5.9	−3.28
30	TDIIDG	6	24.25	Non	−5.8	0.15
31	KKQLM	5	20.39	Non	−5.8	−1.12
32	MQVTPASA	8	22.86	Non	−5.8	0.39

**Table 3:** Molecular docking sites of AHYYD, KPIWT, and TFSGNYP with tyrosinase (2Y9X).

Peptides	Hydrogen Bonds	Hydrophobic Interaction	Electrostatic Interaction
AHYYD	Ser282,Cys83 Glu322,Asn260	Val283,Pro284,His263	
KPIWT	Met280,Asn81	Ala286,His263Val283,Pro284	
TFSGNYP	Asn81,Ser282,Gly281,Val283,Ala246,Gly245	His263,Ala286,Pro284	

**Table 4:** Effects of AHYYD, TFSGNYP, KPIWT, and positive control Kojic acid on tyrosinase activity.

Samples	IC_50_ (mM)
AHYYD	2.012 ± 0.088
TFSGNYP	>5
KPIWT	>10
Kojic acid	0.01 ± 0.003

## Data Availability

The data shown in this study are contained within the article.
